# Simple Power and Sample Size Estimation for Non-Randomized Longitudinal Difference in Differences Studies

**Published:** 2018-11-26

**Authors:** Yirui Hu, DR Hoover

**Affiliations:** 1 Biomedical and Translational Informatics, Geisinger, Danville, 17821, USA; 2 Department of Statistics and Biostatistics and the Institute for Health, Health Care Policy and Aging Research, Rutgers University, Piscataway, 08854, USA

**Keywords:** Non-randomized longitudinal study, Repeated measure, Compound symmetry covariance matrix, Optimal pre-post intervention allocation

## Abstract

Intervention effects on continuous longitudinal normal outcomes are often estimated in two-arm pre-post interventional studies with b≥1 pre- and k≥1 post-intervention measures using “Difference-in-Differences” (DD) analysis. Although randomization is preferred, non-randomized designs are often necessary due to practical constraints. Power/sample size estimation methods for non-randomized DD designs that incorporate the correlation structure of repeated measures are needed. We derive Generalized Least Squares (GLS) variance estimate of the intervention effect. For the commonly assumed compound symmetry (CS) correlation structure (where the correlation between all repeated measures is a constant*ρ*) this leads to simple power and sample size estimation formulas that can be implemented using pencil and paper. Given a constrained number of total timepoints (T), having as close to possible equal number of pre-and post-intervention timepoints (b=k) achieves greatest power. When planning a study with 7 or less timepoints, given large *ρ*(*ρ*≥0.6) in multiple baseline measures (b≥2) or *ρ*≥0.8 in a single baseline setting, the improvement in power from a randomized versus non-randomized DD design may be minor. Extensions to cluster study designs and incorporation of time invariant covariates are given. Applications to study planning are illustrated using three real examples with T=4 timepoints and *ρ* ranging from 0.55 to 0.75.

## Background

Medical intervention studies of chronic conditions and other ongoing processes often evaluate repeated measures of continuous normal outcomes on persons, facilities or other units at systematic timepoints before and after an intervention is delivered [1]. Some of the units are chosen to receive the [1]. Some of the units are chosen to receive the intervention which starts at the same time in all of those units. While randomization of which units (i.e. either individual or facility) receive the intervention is preferred, it is not always feasible; particularly in health economics and services research. Difference-in-Differences (DD) designs thus often estimate impact of a new intervention or policy introduced at a given timepoint for non-randomized treating facilities (or individuals), compared to controls continuing on the existing regimen or policy [2–4]. The outcome being affected by the intervention is measured at *b* consecutive timepoints (enumerated *−b, −(b-1),…. −1*) prior to and *k*. The difference in outcome(s) for the intervention arm between the *b* pre- and *k* post-intervention periods is compared to the corresponding difference for the control arm.

Now DD analysis is best applied using a mixed model framework that adjusts for serial correlation of repeated measures within the same intervention facility or individual [1]. We assume “Non-Randomized” allocation to intervention and control arms being done by convenience or some other process that is not purposely based on levels of the outcome over the first *b* timepoints. For example, maybe hospitals that are closer to a university are assigned the intervention developed at university. Still the pre-intervention levels of the outcome may differ by an unknown amount between the intervention arms due to the criteria that the circumstance allocation was based on even though the investigator was not deliberately seeking for this to happen. For example, perhaps for historical reasons, the outcome measure tends to be higher at those hospitals that were closer to the university. This pre-existing baseline difference between the arms over the *b* pre-intervention timepoints will carry through to any post intervention effect during the *k* post-intervention timepoints. However, as discussed later if the investigator deliberately over selects individual hospitals specifically based on having higher or lower outcome level then the methods covered here do not apply.

In evaluating intervention effect studies, it is important to estimate whether one has a large enough sample to generate precise results. This is commonly denoted power estimation. For repeated-measure longitudinal studies, the “power” depends on an often-unknown correlation structure between repeated measures of the same unit (which may either be a facility or a person) [5]. Repeated measures within the same unit are typically positively correlated which complicates power estimation as well as statistical analysis compared to the standard setting of independence. While general linear models (GLMs) for both statistical analysis and power estimation are well known [6–8] for randomized studies, less power estimation literature exists for non-randomized studies using Difference-in-Differences analysis. This paper develops a generalized least squares (GLS) power estimation framework for non-randomized DD studies using the commonly-assumed compound symmetry (CS) correlation structure of repeated measures that leads to simple power and sample size estimation formulas.

The paper is organized as follows: We first review the standard hypothesis testing and power estimation approach. Next a general linear model of non-randomized pre-post interventional studies with repeated measures using the Difference-in-Differences estimator is presented that develops a standard Generalized Least Squares variance estimate of the intervention effect to be incorporated into the standard power estimation approach. Under the common assumption of compound symmetry repeated-measure correlation, a simple GLS variance formula for intervention effect is derived for non-randomized Difference-in-Differences studies. The influence of number or pre- and post-intervention delivery measures on this variance (and thus efficiency of the study) is evaluated based on this formula. The relative efficiency of non-randomized design is then compared to the randomized setting in terms of needed sample size to achieve the same power. These methods are extended to cluster study designs and incorporation of time invariant covariates. Finally, applications to some recent examples are presented.

## Methods

### General power estimation framework

We consider *H*_*o*_: *θ*=0 versus *H*_*A*_: *θ*=*θ*_*A*_0 where *θ*_*A*_ is some expected or hypothesized value for the intervention effect we wish to statistically detect. Without loss of generality, $\delta =\frac{\theta_{A}}{\sigma }$ is the effect size [9] or *θ*_*A*_ expressed as units of standard deviation. For practical repeated-measure designs, the normal approximation of the non-central *t* distribution can be applied [10]. In specific, the two distributions are almost identical when degrees of freedom (DF) γ>*30* and we have the following equations of power (1−*β*) in eqn. (1), in which $Var\;(\hat{\theta })|$ expressed as GLS variance estimate.

(1) $$\theta_{A}=\left(z_{1-\frac{\alpha }{2}}+z_{1-\beta }\right)\sqrt{Var(\hat{\theta })}.$$

where *α* and *β* are Type I and Type II errors, respectively.

It should be noted here that for smaller sample sizes, it might be appropriate to approximate degrees of freedom in the non-central *t* distribution for the mixture variance (for example, by Satterthwaite’s [11], and Kenward-Roger’s approximations [12]) and adjust (1) for this. But while the full details are beyond the scope of this paper, such will typically not be needed in practice. We now proceed to the derivation of $\;Var\;(\hat{\theta })$ for the Difference-in-Differences design within the General Linear Model Framework.

### General linear model (GLM)

For non-randomized pre-post interventional studies with two intervention arms, researchers encounter repeated measures of a quantitative outcome at *T*=*b+k* systematic timepoints with *b* being before and *k* being after the intervention is delivered to one of the arms. Let *h* denote the intervention arm with *h=0* for control and *h=1* for the new intervention. For each arm, there are *n*_*h*_ units (*n*_*o*_for the control and *n*_*1*_ for the new intervention) and *j=*{−*b*, −(*b*−1),…, −1, 1, 2,…, *k*} denotes the ordered times with {−*b*, *−(b−1)*,…, −1} prior to and {1, 2,…, *k*} being after the intervention onset. The goal is to assess the impact of the new intervention (versus control) on pre-post change in a longitudinal continuous outcome *Y* where *Y*_*1ij*_ is measure *j* from unit *i* in the new intervention arm and *Y*_0i’,_ *j* ‘is measure *j’* from unit *i’* in the control arm. For example, consider a non-randomized trial with *n*_*0*_=*n*_1_=30 hospitals in each arm. Let *i* denote hospitals (as “units”) where *i=1, …,n*_*h*_. The “units” are measured annually for *T=*7 years total with *b*=2 years (2001 to 2002) before and *k*=5 (2003 to 2007) after the intervention implementation in the intervention arm (*h=1*). The outcome of interest, *Y*, could be portion of patients discharged within 30 days after surgery. Thus, *Y*_1,3,−2_ and *Y*_0,17,3_’, respectively, denote the measurement taken in 2001 (2 years prior to start of the intervention) in the 3^*rd*^ hospital of the intervention arm and 2005 (3 years after the start of the intervention) in the 17^*th*^ hospital of the control arm, respectively. We assume complete data with *T=b+k* measures observed on each unit; *Y*_*hij*_can be decomposed as:

(2) $$Y_{hij}=\alpha +\gamma I_{\left\{h=1\right\}}+\beta_{j}+\theta I_{\left\{h=1,j>0\right\}}+\varepsilon_{ij}$$

Now (*α*) is an intercept, which corresponds to the centrality of the control arm. The fixed effect (*γ*) is the difference between the main effect of the intervention and control arms due to non-randomized selection as discussed earlier. The fixed time effect (*β*_*j*_) is modeled to allow for temporal effects at timepoint *j* that are common to both arms.

We assume an immediate impact of size *θ* (i.e., as the DD effect) on the outcome variable for the intervention arm after the intervention begins at time *j*=1 that remains unchanged at subsequent timepoints, which is captured in eqn. (2) by *I*_{h=1,j>0}_ as the intervention effect (*θ*) only delivers to the intervention arm (*h*=1) on the *k* post-intervention measurements. This is an intervention by time interaction mediated by the intervention effect after the it begins. Note that other functions such as linear intervention effect increase *j*∗*θZ*_*hj*_ for *j*≥1 or immediate post-intervention jump followed by exponential decay e^−*j*^ *θZ_*hj*_ for *j* ≥ 1 are possible. However, there are settings where an immediate “jump effect” that continues forward unchanged is appropriate, such as when the intervention is a process change at a medical facility that can be implemented quickly, a drug that the body does not develop resistance or acclimation to, or an immediately successful behavioral intervention. Even if the intervention impact is not “immediate jump”, it could be close to this.

Any random unit (*i*^*th*^level) effects are subsumed into the within-unit error term ε_*ij*,_ where ε_*ij*_~N(0,σ^2^V) with the correlation matrix *V* defined below in eqn. (3). But related to the second paragraph of the Background section, another important assumption on the error term is that of endogenicity; ε_*ij*_ must be independent of the covariates in the model [13]. In this case the covariates are the intercept, indicator for assignment to the intervention arm, indicator of timepoint and the intervention by timepoint interactions. After collection of the data (but not during study planning) there are tests for whether endogenicity problems exist in the data [13]. Still, it is difficult to think of when endogenicity would not hold for our setting with one important exception that was noted earlier. If pre-intervention levels of the outcome are used to identify which units receive the intervention, for example, if poorly performing units or alternately those performing well (i.e. at timepoints *j*<0) are individually over-selected for the intervention, this will likely create endodenocity where ε_*ij*_ for *j*<0 is correlated with the intervention arm assignment. Then issues of regression to the mean [14] destabilize the analyses as the ε_*ij*_ for *j*<0 are not correlated or are less correlated with intervention arm assignment.

The analyses described here using DD estimators are generally not tenable when selection for intervention arm assignment is deliberately based on observed performance of the unit. It is, however, acceptable if better or poorer performing units are placed into one of the arms by circumstance as long as the selection criteria are based on the strata these units fell into being overall better or poorer performing strata. This selection criteria would be independent of ε_*ij*_ and would therefore be captured by inclusion of γ*I*_*{h=1}*_ in eqn. (2).

### Generalized least squares (GLS) estimate

The matrix form of eqn. (2) is: ***Y*** = ***X******β******+ε***, where ε_*ij*_~*N*(0,*σ*^2^*V*). Here *X* represents the design matrix and *Y* is a vector of outcomes. For the general parameter vector ***β*** =, (*á* ***β***_−(***b***−1)_…***β***_−1_,***β***_1_ …***β***_***k***_,***γ***,***θ***,), the corresponding design matrix *X* has (*T*+2) columns (*I*,*J*_−(*b*−1)_,…,*J*_−1_,*J*_1_,…, *J*_*k*_,*I*_{*h*=1}_,*I*_{*h*=1,*j*>0}_, with (*n*_0_+*n*_1_)*T* rows per column (Appendix 1). The indicator columns *I*, *I*_{*h*=1}_,*I*_{*h*=1,*j*>0}_ are coded (0, 1) as defined above; *J*_−(*b*−1)_,…,*J*_−1_,*J*_1_,…,*J*_*k*_ are columns corresponding to (*T−*1) independent time coded variables as follows: for *j=*{-(*b-1), -(b-2),…, −1, 1, 2,…k*}, ***J***_*j*_={−1 at time −*b* (reference); 1 at time *j*; and 0 at all other times}. There is no column for *J*−*b* as $\beta_{-b}=-\sum_{j=-(b-1)}^{k}\beta_{j}$ under the fixed-effects constraint $\sum_{j=-b}^{k}\beta_{j}=0$

More details on the full expansion of design matrix are in Appendix 1. The covariance matrix **V** is made up with (*n*_0_+*n*_1_) times block *T* diagonal matrices ***V***_0_***’****s* with all off-block diagonal matrix elements being 0. The error term measures are independent between units, and within-unit correlation structure is invariant between units (i≠*i′*) for any given two timepoints *j* and *j’*(*j*≠*j′*), that is*, ρ*_*i,jj′*_=*ρ*_*i′,jj′*_. Thus,

(3) $$V={\left(\begin{array}{ccc} V_{0} & \cdots & 0\\ \vdots & \ddots & \vdots \\ 0 & \cdots & V_{0} \end{array}\right)}_{\left(n_{0}+n_{1}\right)T},\text{ where }V_{0}={\left(\begin{array}{cccccc} {\tilde{n}}_{11} & {\tilde{n}}_{12} & {\tilde{n}}_{13} & \cdots & {\tilde{n}}_{1,T-1} & {\tilde{n}}_{1,T}\\ {\tilde{n}}_{21} & {\tilde{n}}_{22} & {\tilde{n}}_{23} & \cdots & {\tilde{n}}_{2,T-2} & {\tilde{n}}_{2,T}\\ {\tilde{n}}_{31} & {\tilde{n}}_{32} & {\tilde{n}}_{33} & \cdots & {\tilde{n}}_{3,T-1} & {\tilde{n}}_{3,T}\\ \vdots & \vdots & \vdots & \cdots & \vdots & \vdots \\ {\tilde{n}}_{T-1,1} & {\tilde{n}}_{T-1,2} & {\tilde{n}}_{T-1,3} & \cdots & {\tilde{n}}_{T-1,T-1} & {\tilde{n}}_{T-1,T}\\ {\tilde{n}}_{T,1} & {\tilde{n}}_{T,2} & {\tilde{n}}_{T,3} & \cdots & {\tilde{n}}_{T-1,T} & {\tilde{n}}_{TT} \end{array}\right)}_{T}$$

The within-unit correlation structure (*ρ*_*jj′*_) is often unknown in advance. Typically, correlation for any two timepoints would be monotonically non-increasing with |*j -j’*|, i.e., as the two timepoints are further separated, they will not become more strongly correlated [15–17].

The Generalized Least Squares (GLS) estimate for ***β*** is $\hat{\underline{\beta }}$ is in eqn. (4), which has proven properties of being the best linear unbiased estimator for ***β*** and uniform minimum variance if *Y*_*hij*_ is normally distributed [6] is now given

(4) $$\underline{\hat{\hat{a}}}={\left(X^{\prime }V^{-1}X\right)}^{-1}X^{\prime }V^{-1}Y.$$

The Generalized Least Squares variance of $\hat{\underline{\beta }}$ is **Λ** in eqn. (5); a square matrix of order *T*+1 with the variance of $\hat{\theta }$ the estimated intervention effect being in the last row and last column of **Λ**.

(5) $$\Lambda ={\left(X^{\prime }V^{-1}X\right)}^{-1}\sigma^{2}.$$

## Results (For Compound Symmetry Correlation)

### GLS estimate

As previously noted, one main difficulty in parametric analysis of longitudinal data lies in specifying covariance structure [17,18], i.e. estimating *ρ*_*jj′*_ for *j ≠ j’*, as normative data from historical settings often does not exist or is limited. However, compound symmetry structure (***V***_**CS**_), in which correlations among repeated measures are assumed to be equal within the same unit, is often a reasonable assumption [19–21]. For example, ***V***_**CS**_ is shown below with *T*=7.

$$V_{\text{cs}=}=\left[\begin{array}{ccccccc} 1 & \rho & \rho & \rho & \rho & \rho & \rho \\ \rho & 1 & \rho & \rho & \rho & \rho & \rho \\ \rho & \rho & 1 & \rho & \rho & \rho & \rho \\ \rho & \rho & \rho & 1 & \rho & \rho & \rho \\ \rho & \rho & \rho & \rho & 1 & \rho & \rho \\ \rho & \rho & \rho & \rho & \rho & 1 & \rho \\ \rho & \rho & \rho & \rho & \rho & \rho & 1 \end{array}\right]$$

From here on we assume compound symmetry correlation. Under CS, the GLS variance $Var\;(\hat{\theta })$ in eqn. (5) as shown in Appendix 2 has a simple form of eqn. (6).

(6) $$Var\;(\hat{\theta })=\left(\frac{1}{n_{0}}+\frac{1}{n_{1}}\right)\frac{T(1-\rho )}{bk}\sigma^{2}=\left(\frac{1}{n_{0}}+\frac{1}{n_{1}}\right)\left(\frac{1}{b}+\frac{1}{\text{k}}\right)(1-\rho )\sigma^{2}.$$

We can then plug in the $Var\;(\hat{\theta })$ in eqn. (6) into eqn. (1) to obtain the number of units in each of the two arms for a given power 1−*β*. For example, if *n*_o_=*n*_1_=*n*, then

(7) $$n=\frac{T(1-\rho )}{bk\delta^{2}}{\left(z_{1-\frac{\alpha }{2}}+z_{1-\beta }\right)}^{2}=\left(\frac{1}{b}+\frac{1}{\text{k}}\right)\frac{\left(1-\rho \right)}{\delta^{2}}{\left(z_{1-\frac{\alpha }{2}}+z_{1-\beta }\right)}^{2}.$$

The next three sections use eqns. (6) and (7) to identify optimal Difference-in-Differences designs, evaluate relative efficiency of non-randomized to randomized designs, and extend to both non-randomized cluster designs and inclusion of additional time invariant covariates into the model. The last section presents applications using derived formulas in three representative examples.

### Optimal pre-post intervention allocation of timepoints

The relatively simple form of eqn. (6), simplifies investigation on optimal pre-post intervention allocation in planning non-randomized DD studies. For example, a repeated-measure design may have a constrained total number of timepoints *T* (*T*=*b* + *k*) because of limited budget and/or time. In such scenarios, finding the optimal allocation of *T* into *b* and *k* that maximizes power (or minimizes the sample size needed to obtain a given power) is important. From eqns. (6) and (7), for CS structure with constrained *T* given *ρ*, the optimal b^*^ with the local minimization of variance is given when *bk*=*b*(*T*−*b*) is maximized.

This occurs at b^*^; If *T* is even, then $b^{*}=k^{*}=\frac{T}{2}$; and if *T* is odd, then equally $b^{*}=\frac{T-1}{2}$ *or* $\frac{T+1}{2}$

**Figure 1:**
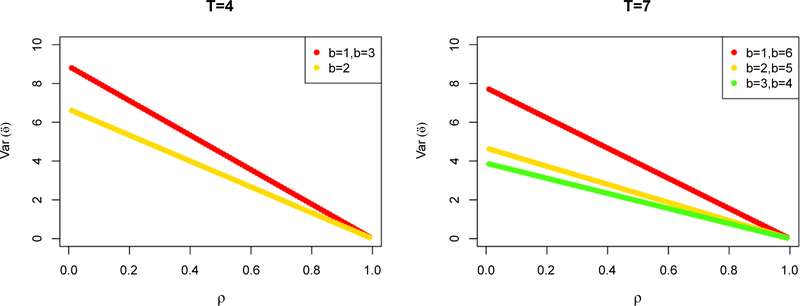
Variance of intervention effect estimate in non-randomized designs under compound symmetry by *b* and *ρ* when *T=*4 and *T=*7.

Figure 1 presents the GLS variance estimates of the intervention effect by the pre-post intervention timepoints allocation and *ρ* (assumingσ^2^=100) for *T=4, T=7*, respectively. From the previous discussion, the optimal b^*^ that minimizes the GLS variance in eqn. (6) is b^*^=2 for *T=4*, and b^*^=3 or 4 for *T=7*. While in common practice, *b=1* is chosen to get units shifted onto intervention as soon as possible, delaying this switch by having multiple pre-intervention timepoints (e.g. *b=2* or *b=3* when *T=7*) substantially reduced the GLS variance estimate of the intervention effect.

### Comparison between the randomized and non-randomized setting

While it is known that randomization is superior to non-randomization, as randomized studies may be more costly and difficult to conduct, the relative superiority may be important to know. Earlier work [19,22] have shown that for a randomized study conducted using the commonly assumed compound symmetry correlation (as shown in Appendix 2).

(8) $$Var\;\left({\hat{\theta }}_{R}\right)=\left(\frac{1}{n_{0}}+\frac{1}{n_{1}}\right)\frac{\left[1+(T-1)\rho ](1-\rho \right)}{k[1+(b-1)\rho ]}\sigma^{2}.$$

To compare eqn. (8) to eqn. (6), we must first address the impact that non-randomization has on *ρ* and σ^2^. For any given setting, σ^2^ will be larger while *ρ* will be smaller in a randomized design as variance about a common global mean due to randomization will be larger than variance about different intervention arm means in the non-randomized design. Compared to the randomized setting with a common intercept, non-randomized setting will result in a lower within population variance on *Y*_*hij*_ where $\sigma^{2}=\sigma_{NR}^{2}<\sigma_{R}^{2}$, together with a smaller within-unit correlation of *Y*_*hij*_ and *Y*_*hij*_ where *ρ*=*ρ*_NR_<*ρ*_R_, due to elimination of variance (_h_) from the total variance. Definitions of $\sigma_{NR}^{2}$, $\sigma_{R}^{2}$, *ρ*_*NR*,_ *ρ*_*R*_ and $\sigma_{e}^{2}$ are presented in Appendix 3. However, from eqn. (6) the $Var\;(\hat{\theta })$ for the non-randomized design only depends on σ^2^ and *ρ* through the product (1−)σ^2^. To that end, this product is unchanged by application of the non-randomized setting in that $\left(1-\rho_{NR}\right)\sigma_{NR}^{2}=\left(1-\rho_{R}\right)\sigma_{R}^{2}=\sigma_{e}^{2}$. This invariance property means that the same effect parameters σ^2^ and *ρ* chosen for a randomized design can be directly used in eqn. (6) to estimate the variance of the intervention effect for the non-randomized designs no matter what the impact non-randomization has on the final σ^2^ and *ρ* is.

To quantitatively measure the difference between randomized and non-randomized studies, we first calculate the ratio of the variance estimate under CS assumption using eqns. (6) and (8) with randomized setting as a reference where as shown above *ρ* is taken from the randomized setting.

(9) $$\frac{\;Var\;(\hat{\theta })}{\;Var\;\left({\hat{\theta }}_{R}\right)}=\frac{T[1+(b-1)\rho ]}{b[1+(T-1)\rho ]}=1+\frac{k(1-\rho )}{b[1+(T-1)\rho ]}=1+\frac{k}{b}*\frac{\left(1-\rho \right)}{\left[1+(T-1)\rho \right]}>1.$$

As *ρ*→1, the ratio goes to 1, meaning the randomized design behaves similar but still better than the non-randomized design when *ρ* is close to 1. As *ρ*→0, the ratio reduces to $1+\frac{k}{b}$, meaning the non-randomized setting requires $\left(1+\frac{k}{b}\right)$ times more units than the comparable randomized setting to achieve the same power when *ρ* is close to 0. Thus, increasing *k* or decreasing *b* (with all other parameters fixed) can lead to more advantages in conducting randomization. For *b*≥*k*, the ratio lies within (1, 2); for very small $\frac{k}{b}$, the ratio is close to 1, meaning that randomization does not qualitatively reduce the GLS variance estimate of the intervention effect.

**Figure 2:**
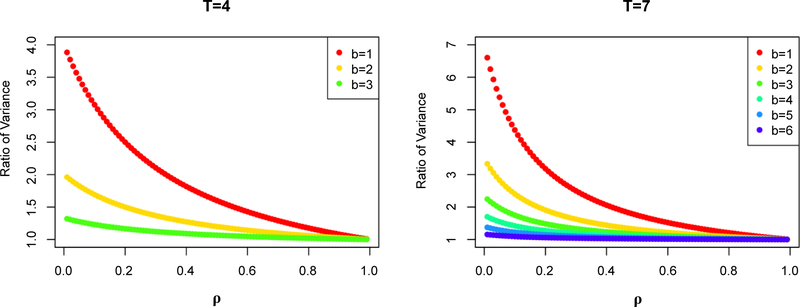
Ratio of the variance of intervention effect estimate non-randomized/randomized design under compound symmetry by *b* and *ρ* for the randomized design when *T=*4 and *T=*7.

Figure 2 provides examples with the number of pre-intervention timepoints (*b*) varying between 1 and (*T*−1) for *T*=4 and *T=7*. We chose *T* to be 4 and *7* as this seems reasonable range for the three examples presented later and other settings where studies would be conducted over periods of 2–4 years with repeated measures at 3–6 month intervals. When *T*=4, for *ρ*≥0.6 and for *b*≥2, non-randomization performed close to randomization as the variance ratio was less than 1.14. But for *b*=1, variance from non-randomization did not approach that from randomization until *ρ*≥0.8 where the variance ratio was 1.17. Similarly, when *T*=7, non-randomization performed close to randomization as the variance ratio was less than 1.22 (for *ρ*≥0.6 and *b*≥2), while variance from non-randomization did not approach that from randomization until *ρ*≥0.8 for *b*=1 where the variance ratio was 1.21.

### Extension to cluster designs

The cluster-randomized trial, with a randomly chosen subset of communities or other units being longitudinally followed that switched into a new intervention at the same timepoint [23], is similar to the pre-post interventional study we have discussed above. However, it differs in that the outcome of the cluster design is not measured on the entire unit, which instead is taken as an average of the outcomes of *m* randomly chosen participants at each new timepoint [23]. For example, *m*=50 *new* participants at the same community are randomly chosen at each timepoint of a smoking cessation intervention study (where all participants at a community receive the same intervention) and the outcome is the average number of cigarettes smoked among these 50 participants. While typically units are randomized into such pre-post intervention studies where *b*=0, *k*=1, repeated-measure designs (*b*>0, *k*>0) with non-randomly chosen units are possible, where *m* different participants being randomly selected within each of all *T* timepoints. Now the outcome is ${\bar{Y}}_{ij}$ an average of *m* independent participants in *i*^*th*^ cluster at time *j*. We should caution the readers that our notation for the covariance *ρ* differs from that used in such cluster designs which we denote as $\tilde{\rho }$ as the variance of the ${\bar{Y}}_{ij}$ depends on *m*. To convert the within-unit repeated-measure correlation $\tilde{\rho }$ used in those papers to our *ρ* defined above, we implement $\rho =\frac{\tilde{p}}{\tilde{\rho }+\frac{1-\tilde{\rho }}{m}}$. Therefore, in a cluster design with *m*=50 participants per cluster where the within-cluster correlation of the outcome is $\tilde{p}$ = 0.02, then the within-unit correlation of the outcome ${\bar{Y}}_{ij}$ from different timepoints (*j* and *j’*) can be derived using the above formula where $\rho =\frac{\tilde{\rho }}{\tilde{\rho }+\frac{1-\tilde{\rho }}{m}}=\frac{0.02}{0.02+\frac{1-0.02}{20}}=0.51.$

### Inclusion of time-invariant covariates

We extend the GLS variance estimate in eqns. (6) and (8) by adding time invariant baseline covariates *W*^1^,…,*W*^*Q*^ (either policy-level or unit-level covariates) to eqn. (2), which can control for compositional changes and further improve the accuracy by decreasing the unmodeled variance. We again assume endogenicity that the added covariates are not correlated with the error term [13,24]. While this assumption must always be evaluated for any given setting, it is difficult to see how external baseline covariates could be correlated with *ε*_*ij*_ except perhaps through interaction with the previously described over selection of poorly (or well) performing units at *j*−1 to be put into the intervention arm. We noted before, that after collection of the data there are tests for whether endogenicity problems exist [13]. As derived in Appendix 4, if endogneicity problems do not exist, then after inclusion of time invariant covariate into the model.

(10) $$Var\;(\hat{\theta })=\left(\frac{1}{n_{0}}+\frac{1}{n_{1}}\right)\frac{T\left(1-\rho_{Q}\right)}{bk}\sigma_{Q}^{2}=\left(\frac{1}{n_{0}}+\frac{1}{n_{1}}\right)\left(\frac{1}{b}+\frac{1}{\text{k}}\right)\left(1-\rho_{Q}\right)\sigma_{Q}^{2}.$$

where *R*^*2*^ is the multiple correlation coefficient between *W*^1^,…,*W*^*Q*^ and *Y* while *ρ*_*Q*_ and $\sigma_{Q}^{2}$ are the within-unit correlations and variances of Y after adjusting for baseline covariates *W*^1^,…, *W*^q^ and $\sigma_{Q}^{2}=\left(1-R^{2}\right)\sigma^{2}$ where σ^2^ is what the variance would have been without the additional covariates.

### Application to representative examples

We selected three representative examples from available data and literature to evaluate potential repeated-measure correlation studies; i) depression measured by the Center for Epidemiologic Studies Score for Depression (CESD) in women seen every 6 months [25] on which we observed *ρ*=0.55, ii) cardiovascular and other clinical measures of regularly monitored patients for which ~ 0.65 was observed [19], and iii) a longitudinal study of an intervention of community-based sanitation in Indian villages for which *ρ*=0.75 was noted [26].

Let’s assume that for one of these settings, a non-randomized longitudinal study with two intervention arms is planned, for *T*=4. The goal of interest is to calculate the number of units needed to obtain a specified study size and power. In many settings, the intervention would be implemented after one baseline visit (i.e., *b*=1, *k*=3). But from previous discussion of optimal pre-post allocation, the GLS variance of the intervention effect in the non-randomized design (the main focus of this paper) is minimized with *b*=*k*=3. In some settings, *b*=3, *k*=1 might be used. Table 1 thus provides the needed sample size (units for both arms) to detect effect size *δ*=0.25 and *δ*=0.50 as calculated using eqn. (7) at 80% power for two-sided hypothesis testing at 0.05 significance-level. This is done for (*b*,*k*)=(1,3), (2,2) and (3,1) for both non-randomized and randomized setting. For simplicity (and to optimized power), *n*_0_=*n*_1_=*n* is assumed.

For example, when planning a non-randomized DD study, if (*b,k*)=(1,3), *δ*=0.50, *ρ*=0.55, from eqn. (7),

$$n=\frac{T(1-\rho )}{bk\delta^{2}}{\left(z_{1-\frac{\alpha }{2}}+z_{1-\beta }\right)}^{2}=\frac{4*(1-0.55)}{1^{*}3^{*}0.50^{2}}*{\left(1.96+0.84\right)}^{2}\approx 19.$$

Thus, we would need a total sample size of 38 units for both arms to achieve 80% power at 0.05 significance-level.

Now comparing the non-randomized DD to a randomized study, the corresponding randomized setting reduced the number of needed units for both arms by ~17% (70 vs. 84 for *δ*=0.25 and 18 vs. 22 for *δ*=0.50 when (*b,k*)=(1,3) and *ρ*=0.55. But the reduction in needed units for randomized vs. non-randomized setting when (*b,k*)=(1,3) and *ρ*=0.65 was higher, approximately 32%−35% (as 100 vs. 152 for *δ*=0.25 and 26 vs. 38 for *δ*=0.50).

However, the total number of units needed for both the randomized as well as the non-randomized design was lower for (*b,k*)=(2,2) than for (*b,k*)=(1,3) in these examples. For the non-randomized design, the reduction in units needed for (*b,k*)=(2,2) vs. (*b,k*)=(1,3) was ~25%, which follows from b* k being in the denominator of formula (7) with the ratio $\frac{1*3}{2*2}=0.75$ With (*b,k*)=(2,2), the advantage of the randomized vs. non-randomized was much less than it was with (*b,k*)=(1,3), as the total number of units needed ranging from no reduction at all (16 for both when *ρ*=0.75 and *δ*=0.50), to 14% (98 vs. 114 when *ρ*=0.55 and *δ*=0.25).

By symmetry, the total number of units needed for (*b,k*)=(1,3) was the same as for (*b,k*)=(3,1) in the non-randomized design. However, for the randomized design, the total number of units needed was much larger for (*b,k*)=(3,1) than for (*b,k*)=(1,3). Thus, the advantage of randomization over non-randomized was small when (*b,k*)=(3,1), ranging from no reduction in needed units when *ρ*=0.75 and *δ*=0.50, to a reduction of 8 units (~5%) from 152 to 144 for both arms when *ρ*=0.55 and *δ*=0.25.

## Conclusion

The aim of this paper was to develop a power and sample size estimation framework for non-randomized two-arm pre-post interventional studies with repeated continuous longitudinal outcomes using Difference-in-Differences analysis. We presented generalized least squares variance estimates of intervention effect in linear models assuming a jump effect on the outcome immediately after intervention.

An easily implemented formula for variance estimate of intervention effect was derived under the commonly-assumed within-unit compound symmetry correlation among repeated measures. Not surprisingly, the variance decreases as the number of total timepoints (*T*) increases. However, this must be weighed against the extra cost associated with more follow-up timepoints. For non-randomized DD studies with a constrained *T*, equal number of pre- and post-intervention timepoints can achieve the greatest power by minimizing the GLS variance of intervention effect.

For power analysis in planning intervention studies with pre-post intervention outcomes and *T* ranging from 4 to 7, although randomization is always preferred, non-randomization can work nearly as well for high within-unit repeated-measure correlation (*ρ*≥0.60) in multiple baseline designs where *b*≥2, while for single baseline designs where *b*=1, researchers should be more cautious about choosing non-randomization unless a higher correlation (*b*=1, *ρ* ≥ 0.80) exists.

To extend GLS variance formulas to studies conducted with a cluster design approach with the outcome ${\bar{Y}}_{ij}$, being an average of *m* different participants randomly chosen from the cluster we convert the $\tilde{\rho }$ used for the “within-cluster correlation” in those papers to our within-unit repeated-measure correlation *ρ* using $\rho =\frac{\tilde{\rho }}{\tilde{\rho }+\frac{1-\tilde{\rho }}{m}}$. We further extended GLS variance formulas by incorporating time-invariant covariates, that can reduce the variance of the intervention effect estimate by (1−*R*^2^) where *R*^2^ is the multiple correlation coefficient between those covariates and the outcome.

Several limitations should be mentioned. For simplicity, we focused on designs with no missing data, although such will likely be the case when units are facilities with the outcome data collected by ongoing periodic quality control monitoring even in the absence of a DD study. We assumed an immediate one-time jump effect of the intervention. While the effect may have accruing cumulative or some other patterns in some settings, it may still be very close to an immediate jump. Although compound symmetry correlation is often assumed when planning a study, it may not always hold in practice as covariance could change over time from uncontrollable mechanisms. Relaxation of the above assumptions may likely lead to complicated settings that could be addressed with simulation.

In conclusion, this paper developed a generalized least squares power estimation framework based on compound symmetry correlation that resulted in simple GLS variance formulas of the intervention effect for non-randomized Difference-in-Differences studies which could be implemented with pencil and paper. We investigated the optimal pre-post intervention allocation of timepoints in planning non-randomized longitudinal Difference-in-Differences studies. While randomization is always preferred to reduce the variance estimate of the intervention effect, non-randomization performs relatively well (for *T*≤7 timepoints) when high within-unit repeated-measure correlation holds particularly if there is a large number of pre-intervention relative to post-intervention timepoints. The formulas easily extend to cluster study designs and adjust for time invariant variables.

## Supplementary Material

Appendix 1-4

## Figures and Tables

**Table 1:** Total sample size needed for 80% power at 0.05 level for settings with *ρ* ranging from 0.55 to 0.75 when *n*^0^=*n*^1^=*n*.

	δ=0.25		δ=0.50	
	Randomized	Non-Randomized	Randomized	Non-Randomized
		*ρ*=0.55		
(*b,k*)=(1,3)	100	152	26	38
(*b,k*)=(1,3)	98	114	26	30
((*b,k*)=(1,3)	144	152	36	38
		*ρ*=0.60		
(*b,k*)=(1,3)	94	134	24	34
(*b,k*)=(1,3)	88	102	22	26
(*b,k*)=d,3)	128	134	32	34
		*ρ*=0.75		
(*b,k*)=(1,3)	70	84	18	22
(*b,k*)=(1,3)	60	64	16	16
(*b,k*)=(1,3)	82	84	22	22

## Abbreviations:

DD: Difference-in-Differences
CS: Compound Symmetry
GLM: General Linear Model
GLS: Generalized Least Squares
