# Supplementary material for: Simple Power and Sample Size Estimation for Non-Randomized Longitudinal Difference in Differences Studies
Source: J Biom Biostat. Author manuscript; Available in PMC 2019 Jul 29. (PMC6663085)
Supplement: Appendix 1-4 [file NIHMS1040609-supplement-Appendix_1-4.pdf]

APPENDIX 1: DESIGN MATRIX AND COMPOUND SYMMETRY

The design matrix $\boldsymbol{X}$ is made up of $(n_0 + n_1)T$ rows and $(T + 2)$ columns, where $\boldsymbol{X_{h=0,i}}$ represents a unit from an untreated (or control) arm; $\boldsymbol{X_{h=1,i}}$ stands for a unit that receives the new intervention. The first T columns stand for the intercept and time effects as defined in the main paper, the $(T + 1)^{th}$column indicates a long-term non-randomization effect, and the $(T + 2)^{th}$column represents the intervention effect. Note that the design matrix reduces to randomized design if the $(T + 1)^{th}$ column is left out of the design matrix $\boldsymbol{X}$.

$$\boldsymbol{X} = \begin{bmatrix} \boldsymbol{X_{h=0,1}} \\ \vdots \\ \boldsymbol{X_{h=0,n_0}} \\ \boldsymbol{X_{h=1,1}} \\ \vdots \\ \boldsymbol{X_{h=1,n_1}} \end{bmatrix} = [\boldsymbol{X_T} \quad \boldsymbol{X_2}]$$ where $\boldsymbol{X_T}$ is the first T columns, $\boldsymbol{X_2}$ is the last two columns, and

$$\boldsymbol{X_{h=0,i}} = \begin{bmatrix} \mathbf{1} & \mathbf{-1} & \cdots & \mathbf{-1} & \mathbf{-1} & \cdots & \mathbf{-1} & -\boldsymbol{n_1}/(\boldsymbol{n_0}+\boldsymbol{n_1}) & \mathbf{0} \\ \mathbf{1} & \mathbf{1} & \cdots & \mathbf{0} & \mathbf{0} & \cdots & \mathbf{0} & -\boldsymbol{n_1}/(\boldsymbol{n_0}+\boldsymbol{n_1}) & \mathbf{0} \\ \vdots & \vdots & \ddots & \vdots & \vdots & \ddots & \vdots & \vdots & \vdots \\ \mathbf{1} & \mathbf{0} & \cdots & \mathbf{1} & \mathbf{0} & \cdots & \mathbf{0} & -\boldsymbol{n_1}/(\boldsymbol{n_0}+\boldsymbol{n_1}) & \mathbf{0} \\ \mathbf{1} & \mathbf{0} & \cdots & \mathbf{0} & \mathbf{1} & \cdots & \mathbf{0} & -\boldsymbol{n_1}/(\boldsymbol{n_0}+\boldsymbol{n_1}) & -\boldsymbol{n_1}/(\boldsymbol{n_0}+\boldsymbol{n_1}) \\ \mathbf{1} & \mathbf{0} & \cdots & \mathbf{0} & \mathbf{0} & \cdots & \mathbf{0} & -\boldsymbol{n_1}/(\boldsymbol{n_0}+\boldsymbol{n_1}) & -\boldsymbol{n_1}/(\boldsymbol{n_0}+\boldsymbol{n_1}) \\ \vdots & \vdots & \ddots & \vdots & \vdots & \ddots & \vdots & \vdots & \vdots \\ \mathbf{1} & \mathbf{0} & \cdots & \mathbf{0} & \mathbf{0} & \cdots & \mathbf{1} & -\boldsymbol{n_1}/(\boldsymbol{n_0}+\boldsymbol{n_1}) & -\boldsymbol{n_1}/(\boldsymbol{n_0}+\boldsymbol{n_1}) \end{bmatrix}_{\boldsymbol{T*(T+2)}},$$

$$\boldsymbol{X_{h=1,i}} = \begin{bmatrix} \mathbf{1} & \mathbf{-1} & \cdots & \mathbf{-1} & \mathbf{-1} & \cdots & \mathbf{-1} & \boldsymbol{n_0}/(\boldsymbol{n_0}+\boldsymbol{n_1}) & \mathbf{0} \\ \mathbf{1} & \mathbf{1} & \cdots & \mathbf{0} & \mathbf{0} & \cdots & \mathbf{0} & \boldsymbol{n_0}/(\boldsymbol{n_0}+\boldsymbol{n_1}) & \mathbf{0} \\ \vdots & \vdots & \ddots & \vdots & \vdots & \ddots & \vdots & \vdots & \vdots \\ \mathbf{1} & \mathbf{0} & \cdots & \mathbf{1} & \mathbf{0} & \cdots & \mathbf{0} & \boldsymbol{n_0}/(\boldsymbol{n_0}+\boldsymbol{n_1}) & \mathbf{0} \\ \mathbf{1} & \mathbf{0} & \cdots & \mathbf{0} & \mathbf{1} & \cdots & \mathbf{0} & \boldsymbol{n_0}/(\boldsymbol{n_0}+\boldsymbol{n_1}) & \boldsymbol{n_0}/(\boldsymbol{n_0}+\boldsymbol{n_1}) \\ \mathbf{1} & \mathbf{0} & \cdots & \mathbf{0} & \mathbf{0} & \cdots & \mathbf{0} & \boldsymbol{n_0}/(\boldsymbol{n_0}+\boldsymbol{n_1}) & \boldsymbol{n_0}/(\boldsymbol{n_0}+\boldsymbol{n_1}) \\ \vdots & \vdots & \ddots & \vdots & \vdots & \ddots & \vdots & \vdots & \vdots \\ \mathbf{1} & \mathbf{0} & \cdots & \mathbf{0} & \mathbf{0} & \cdots & \mathbf{1} & \boldsymbol{n_0}/(\boldsymbol{n_0}+\boldsymbol{n_1}) & \boldsymbol{n_0}/(\boldsymbol{n_0}+\boldsymbol{n_1}) \end{bmatrix}_{\boldsymbol{T*(T+2)}}.$$

For the compound symmetry setting with $\rho_{jj'}$'≡ ρ where the correlation matrix is $\boldsymbol{V} = \begin{pmatrix} \boldsymbol{V_0} & \cdots & \mathbf{0} \\ \vdots & \ddots & \vdots \\ \mathbf{0} & \cdots & \boldsymbol{V_0} \end{pmatrix}_{(\boldsymbol{n_0}+\boldsymbol{n_1})\boldsymbol{T}}$ where $\boldsymbol{V_0} = \begin{pmatrix} \mathbf{1} & \cdots & \boldsymbol{\rho} \\ \vdots & \ddots & \vdots \\ \boldsymbol{\rho} & \cdots & \mathbf{1} \end{pmatrix}_{\boldsymbol{T}}$. Thus, $\boldsymbol{V^{-1}} = \begin{pmatrix} \boldsymbol{V_0}^{-1} & \cdots & \mathbf{0} \\ \vdots & \ddots & \vdots \\ \mathbf{0} & \cdots & \boldsymbol{V_0}^{-1} \end{pmatrix}_{(\boldsymbol{n_0}+\boldsymbol{n_1})\boldsymbol{T}}$ where $\boldsymbol{V_0}^{-1} =$

$$\frac{\mathbf{1}}{[\mathbf{1}+(\boldsymbol{T}-\mathbf{1})\boldsymbol{\rho}](\mathbf{1}-\boldsymbol{\rho})} \begin{pmatrix} \mathbf{1}+(\boldsymbol{T}-\mathbf{2})\boldsymbol{\rho} & \cdots & -\boldsymbol{\rho} \\ \vdots & \ddots & \vdots \\ -\boldsymbol{\rho} & \cdots & \mathbf{1}+(\boldsymbol{T}-\mathbf{2})\boldsymbol{\rho} \end{pmatrix}_{\boldsymbol{T}}.$$

APPENDIX 2: GLS estimate under CS

To derive the GLS variance $Var(\hat{\theta})$, i.e., the last element of $(\boldsymbol{X'V^{-1}X})^{-1}\sigma^2$, we applied the technique using inverse of the partitioned matrix. For simplicity, we will illustrate with $n_0 = n_1 = n$ and partition $(\boldsymbol{X'V^{-1}X})$ into $\mathbf{A}_{11}$ the first T columns and rows, $\mathbf{A}_{21}$ the last two rows and first T columns, $\mathbf{A}_{12}$ the last two columns and first T rows and $\mathbf{A}_{22}$ the last two rows and columns, then derive $(\boldsymbol{X'V^{-1}X})^{-1} = [1 + (T-1)\rho](1-\rho)\begin{bmatrix} \boldsymbol{A_{11}} & \boldsymbol{A_{12}} \\ \boldsymbol{A_{21}} & \boldsymbol{A_{22}} \end{bmatrix}^{-1}$.

Note that $\boldsymbol{A_{21}} = \boldsymbol{A'_{21}} = \boldsymbol{X'_T V^{-1} X_2} = \begin{bmatrix} \mathbf{0} & \mathbf{0} \\ \vdots & \vdots \\ \mathbf{0} & \mathbf{0} \end{bmatrix}_{\boldsymbol{T*2}}$ as the positive and negative terms in this multiplication of matrices cancel out and $\boldsymbol{A_{22}} = \boldsymbol{X'_2 V^{-1} X_2} = \frac{n(1-\rho)}{2}\begin{bmatrix} \boldsymbol{T} & \boldsymbol{k} \\ \boldsymbol{k} & \frac{\boldsymbol{k[1+(b-1)\rho]}}{\boldsymbol{(1-\rho)}} \end{bmatrix}$. We can apply the technique for the inverse of the partitioned matrix, i.e., $\begin{bmatrix} \boldsymbol{A_{11}} & \boldsymbol{A_{21}} \\ \boldsymbol{A_{21}} & \boldsymbol{A_{22}} \end{bmatrix}^{-1} = \begin{bmatrix} \boldsymbol{B_{11}} & \boldsymbol{B_{21}} \\ \boldsymbol{B_{21}} & \boldsymbol{B_{22}} \end{bmatrix}$ where $\boldsymbol{B_{22}} = \left(\boldsymbol{A_{22}} - \boldsymbol{A_{21}A_{11}}^{-1}\boldsymbol{A_{12}}\right)^{-1}$. Since $\boldsymbol{A_{21}} = \boldsymbol{A'_{12}} = \begin{bmatrix} \mathbf{0} & \mathbf{0} \\ \vdots & \vdots \\ \mathbf{0} & \mathbf{0} \end{bmatrix}$, $\boldsymbol{A_{21}A_{11}}^{-1}\boldsymbol{A_{12}} = \begin{bmatrix} \mathbf{0} & \mathbf{0} \\ \mathbf{0} & \mathbf{0} \end{bmatrix}$, $\boldsymbol{B_{22}} = \boldsymbol{A_{22}}^{-1} = \frac{2}{n(1-\rho)}\frac{(1-\rho)}{bk[1+(T-1)\rho]}\begin{bmatrix} \frac{\boldsymbol{k[1+(b-1)\rho]}}{\boldsymbol{(1-\rho)}} & \boldsymbol{-k} \\ \boldsymbol{-k} & \boldsymbol{T} \end{bmatrix} = \frac{2}{nbk[1+(T-1)\rho]}\begin{bmatrix} \frac{\boldsymbol{k[1+(b-1)\rho]}}{\boldsymbol{(1-\rho)}} & \boldsymbol{-k} \\ \boldsymbol{-k} & \boldsymbol{T} \end{bmatrix}$.

As noted earlier, $Var(\hat{\theta})$is the most lower right element of $(\boldsymbol{X'V^{-1}X})^{-1}\sigma^2$, i.e., $[1 + (T-1)\rho](1 - \rho)\frac{2T}{nbk[1-(T-1)\rho]}\sigma^2 = \frac{2T(1-\rho)}{nbk}\sigma^2$, where $n_0 = n_1 = n$. Therefore, $Var(\hat{\theta}) = \frac{2T(1-\rho)}{nbk}\sigma^2$. We then derive this simple closed form GLS variance formula assuming CS.

A. Non-Randomized setting

$$Var(\hat{\theta}) = (\frac{1}{n_0} + \frac{1}{n_1})\frac{T(1-\rho)}{bk}\sigma^2.$$

Note that if the $(T+1)^{th}$ column is left out as described in Appendix 1, then A_{22} reduces to a 1 x 1 matrix with the value $\frac{nk[1+(b-1)\rho]}{2}$ using the above approach that results in

B. Randomized setting

$$Var(\hat{\theta}_R) = (\frac{1}{n_0} + \frac{1}{n_1})\frac{[1+(T-1)\rho](1-\rho)}{k[1+(b-1)\rho]}\sigma^2.$$

APPENDIX 3: Under Compound symmetry correlation the ρ and σ^2 from a randomized design can be exported to an embedded non-randomized design

For randomized setting with compound symmetry repeated-measure correlation, $Y_{hij} = \alpha + \beta_j + \theta + \mu_i + e_{ij}$, where the independent random unit effect $\mu_i \sim N(0, \tau^2)$ and the independent within-unit error $e_{ij} \sim N(0, \sigma_e^2)$. The total variance can be decomposed: $\sigma_R^2 = \tau^2 + \sigma_e^2, \rho_R = \frac{\tau^2}{\tau^2+\sigma_e^2}$, and $1 - \rho_R = \frac{\sigma_e^2}{\tau^2+\sigma_e^2}$. We now use the subscript "R" in ρ_R and σ_R^2 to distinguish from the ρ and $\sigma^2$of an embedded non-randomized design.

For non-randomized DD study, $Y_{hij} = \alpha + \beta_j + \theta + \gamma_h + (\mu_i - \gamma_h) + e_{ij}$, where the non-randomization group effect $\gamma_h \sim N(0, \sigma_h^2)$, $(\mu_i - \gamma_h) \sim N(0, \tau^2 - \sigma_h^2)$, $e_{ij} \sim N(0, \sigma_e^2)$. Here we assume that γ_h, μ_i are independent. Note in (2), under the assumption of compound symmetry correlation structure, $\varepsilon_{ij} = e_{ij} + (\mu_i - \gamma_h)$. The intervention arm assignment is carried by γ_h, which results in the variance of unit effect now being $(\mu_i - \gamma_h)$ by eliminating σ_h^2 from both the variance and the within-unit correlation. The total variance can then be decomposed: $\sigma_{NR}^2 = (\tau^2 - \sigma_h^2) + \sigma_e^2$, $\rho_{NR} = \frac{\tau^2-\sigma_h^2}{\sigma_{NR}^2} = \frac{\tau^2-\sigma_h^2}{(\tau^2-\sigma_h^2)+\sigma_e^2}$ and $(1 - \rho_{NR}) = \frac{\sigma_e^2}{(\tau^2-\sigma_h^2)+\sigma_e^2}$. Again, the subscript "NR" has been added to specifically mark the differences of the randomized and non-randomized designs as in general $\sigma_R^2 \neq \sigma_{NR}^2$ and $\rho_R \neq \rho_{NR}$.

The products $(1 - \rho_R)\sigma_R^2$ and $(1 - \rho_{NR})\sigma_{NR}^2$ are, however, equal as follows.

$$\begin{cases} (1 - \rho_R)\sigma_R^2 = \dfrac{\sigma_e^2}{\tau^2 + \sigma_e^2}(\tau^2 + \sigma_e^2) = \sigma_e^2 \\ (1 - \rho_{NR})\sigma_{NR}^2 = \dfrac{\sigma_e^2}{(\tau^2 - \sigma_h^2) + \sigma_e^2}[(\tau^2 - \sigma_h^2) + \sigma_e^2] = \sigma_e^2 \end{cases}$$

In conclusion, $(1 - \rho_{NR})\sigma_{NR}^2 = (1 - \rho_R)\sigma_R^2$. As both (6) and (8) depend on the product $(1 - \rho)\sigma^2$, meaning that for comparisons of the GLS variance estimate of the intervention effect from a randomized design and an embedded non-randomized design under compound symmetry, ρ and $\sigma^2$from the randomized design can be used in (6) through the product $(1 - \rho)\sigma^2$, even though the individual parameters are changed by non-randomization.

APPENDIX 4: Extension to include baseline covariates

Consider Q within-unit time invariant covariates in a matrix $\boldsymbol{W^1}, \dots, \boldsymbol{W^Q}$, each presented in a column vector, and let $\bar{\mu}_0^q$ and $\bar{\mu}_1^q$ be the means of each of these covariates among all n_0 units in the control and n_1 units in the intervention arm respectively for *q = 1, ..., Q*. Again, we assume endogenicity that the covariates are not correlated with the error term. Then from the observed $w_{h,i}^q$ for each unit, create a within intervention arm centered value of that covariate $\widetilde{w}_{h,i}^q = w_{h,i}^q - \bar{\mu}_h^q$ for *q = 1, ..., Q*. Now expand the design matrix in Appendix 1 to include these within intervention arm centered covariates as: $\boldsymbol{X} = \begin{bmatrix} \boldsymbol{X_{h=0,1}} \\ \vdots \\ \boldsymbol{X_{h=0,n_0}} \\ \boldsymbol{X_{h=1,1}} \\ \vdots \\ \boldsymbol{X_{h=1,n_1}} \end{bmatrix} = [\boldsymbol{X_{T+Q}} \quad \boldsymbol{X_2}]$ where $\boldsymbol{X_{T+Q}}$ is the first $(T+Q)$ columns, $\boldsymbol{X_2}$ is the last two columns, and $\boldsymbol{X_{h=0,i}} =$

$$\begin{pmatrix} \boldsymbol{1} & \boldsymbol{-1} & \cdots & \boldsymbol{-1} & \boldsymbol{-1} & \cdots & \boldsymbol{-1} & \boldsymbol{\widetilde{w}_{0,i}^1} & \cdots & \boldsymbol{\widetilde{w}_{0,i}^Q} & -\boldsymbol{n_1/(n_0+n_1)} & \boldsymbol{0} \\ \boldsymbol{1} & \boldsymbol{1} & \cdots & \boldsymbol{0} & \boldsymbol{0} & \cdots & \boldsymbol{0} & \boldsymbol{\widetilde{w}_{0,i}^1} & \cdots & \boldsymbol{\widetilde{w}_{0,i}^Q} & -\boldsymbol{n_1/(n_0+n_1)} & \boldsymbol{0} \\ \vdots & \vdots & \ddots & \vdots & \vdots & \ddots & \vdots & \vdots & \ddots & \vdots & \vdots & \vdots \\ \boldsymbol{1} & \boldsymbol{0} & \cdots & \boldsymbol{1} & \boldsymbol{0} & \cdots & \boldsymbol{0} & \boldsymbol{\widetilde{w}_{0,i}^1} & \cdots & \boldsymbol{\widetilde{w}_{0,i}^Q} & -\boldsymbol{n_1/(n_0+n_1)} & \boldsymbol{0} \\ \boldsymbol{1} & \boldsymbol{0} & \cdots & \boldsymbol{0} & \boldsymbol{1} & \cdots & \boldsymbol{0} & \boldsymbol{\widetilde{w}_{0,i}^1} & \cdots & \boldsymbol{\widetilde{w}_{0,i}^Q} & -\boldsymbol{n_1/(n_0+n_1)} & -\boldsymbol{n_1/(n_0+n_1)} \\ \boldsymbol{1} & \boldsymbol{0} & \cdots & \boldsymbol{0} & \boldsymbol{0} & \cdots & \boldsymbol{0} & \boldsymbol{\widetilde{w}_{0,i}^1} & \cdots & \boldsymbol{\widetilde{w}_{0,i}^Q} & -\boldsymbol{n_1/(n_0+n_1)} & -\boldsymbol{n_1/(n_0+n_1)} \\ \vdots & \vdots & \ddots & \vdots & \vdots & \ddots & \vdots & \vdots & \ddots & \vdots & \vdots & \vdots \\ \boldsymbol{1} & \boldsymbol{0} & \cdots & \boldsymbol{0} & \boldsymbol{0} & \cdots & \boldsymbol{1} & \boldsymbol{\widetilde{w}_{0,i}^1} & \cdots & \boldsymbol{\widetilde{w}_{0,i}^Q} & -\boldsymbol{n_1/(n_0+n_1)} & -\boldsymbol{n_1/(n_0+n_1)} \end{pmatrix}_{\boldsymbol{T*(T+Q+2)}},$$

$$\boldsymbol{X_{h=1,i}} = \begin{pmatrix} \boldsymbol{1} & \boldsymbol{-1} & \cdots & \boldsymbol{-1} & \boldsymbol{-1} & \cdots & \boldsymbol{-1} & \boldsymbol{\widetilde{w}_{1,i}^1} & \cdots & \boldsymbol{\widetilde{w}_{1,i}^Q} & \boldsymbol{n_0/(n_0+n_1)} & \boldsymbol{0} \\ \boldsymbol{1} & \boldsymbol{1} & \cdots & \boldsymbol{0} & \boldsymbol{0} & \cdots & \boldsymbol{0} & \boldsymbol{\widetilde{w}_{1,i}^1} & \cdots & \boldsymbol{\widetilde{w}_{1,i}^Q} & \boldsymbol{n_0/(n_0+n_1)} & \boldsymbol{0} \\ \vdots & \vdots & \ddots & \vdots & \vdots & \ddots & \vdots & \vdots & \ddots & \vdots & \vdots & \vdots \\ \boldsymbol{1} & \boldsymbol{0} & \cdots & \boldsymbol{1} & \boldsymbol{0} & \cdots & \boldsymbol{0} & \boldsymbol{\widetilde{w}_{1,i}^1} & \cdots & \boldsymbol{\widetilde{w}_{1,i}^Q} & \boldsymbol{n_0/(n_0+n_1)} & \boldsymbol{0} \\ \boldsymbol{1} & \boldsymbol{0} & \cdots & \boldsymbol{0} & \boldsymbol{1} & \cdots & \boldsymbol{0} & \boldsymbol{\widetilde{w}_{1,i}^1} & \cdots & \boldsymbol{\widetilde{w}_{1,i}^Q} & \boldsymbol{n_0/(n_0+n_1)} & \boldsymbol{n_0/(n_0+n_1)} \\ \boldsymbol{1} & \boldsymbol{0} & \cdots & \boldsymbol{0} & \boldsymbol{0} & \cdots & \boldsymbol{0} & \boldsymbol{\widetilde{w}_{1,i}^1} & \cdots & \boldsymbol{\widetilde{w}_{1,i}^Q} & \boldsymbol{n_0/(n_0+n_1)} & \boldsymbol{n_0/(n_0+n_1)} \\ \vdots & \vdots & \ddots & \vdots & \vdots & \ddots & \vdots & \vdots & \ddots & \vdots & \vdots & \vdots \\ \boldsymbol{1} & \boldsymbol{0} & \cdots & \boldsymbol{0} & \boldsymbol{0} & \cdots & \boldsymbol{1} & \boldsymbol{\widetilde{w}_{1,i}^1} & \cdots & \boldsymbol{\widetilde{w}_{1,i}^Q} & \boldsymbol{n_0/(n_0+n_1)} & \boldsymbol{n_0/(n_0+n_1)} \end{pmatrix}_{\boldsymbol{T*(T+Q+2)}}.$$

By matrix multiplication, we would have $\boldsymbol{\widetilde{W}^{q\prime}} \boldsymbol{V^{-1}} \boldsymbol{X_2} = [\boldsymbol{0} \quad \boldsymbol{0}]$ where $\boldsymbol{\widetilde{W}^{q\prime}} = (\boldsymbol{\widetilde{w}_{h,i}^q}, \dots, \boldsymbol{\widetilde{w}_{h,i}^q})_{\boldsymbol{1*(n_0+n_1)T}}$. Thus when $\boldsymbol{X_T}$ is expanded to include $\boldsymbol{\widetilde{W}^q}$ (for *q = 1, ..., Q*) by this orthogonal coding $\boldsymbol{X_{T+Q}}' \boldsymbol{V^{-1}} \boldsymbol{X_2} = \begin{bmatrix} \boldsymbol{0} & \boldsymbol{0} \\ \vdots & \vdots \\ \boldsymbol{0} & \boldsymbol{0} \end{bmatrix}_{\boldsymbol{(T+Q)*2}}$.

Therefore, the formulas in Appendix 1 remain unchanged except that by adding the baseline covariates into the model, the residual variance is further reduced to $\sigma_Q^2 = (1 - R^2)\sigma^2$ where R^2 is the multiple correlation coefficient

between $\boldsymbol{W^1}, \ldots, \boldsymbol{W^Q}$ and outcome after adjusting for all other variables in the model, and that the within-unit correlation of repeated measures is now ρ_Q the within-unit correlation after adjusting for these covariates. This leads to the following variances for the intervention effect estimate.

A. Non-Randomized setting

$$Var(\hat{\theta}) = (\frac{1}{n_0} + \frac{1}{n_1})\frac{T(1-\rho_Q)}{bk}\sigma_Q^2$$

B. Randomized setting

$$Var(\hat{\theta}_R) = (\frac{1}{n_0} + \frac{1}{n_1})\frac{[1+(T-1)\rho_Q](1-\rho_Q)}{k[1+(b-1)\rho_Q]}\sigma_Q^2$$

Note that while centering the values of $w_{h,i}^q$ at different means for the control and intervention arms achieves the desired orthogonality, the differential baseline main effect between the arms is now between a control-arm observation with $w_{0,i}^q - \bar{\mu}_0^q$ and an intervention-arm observation with $w_{1,i}^q - \bar{\mu}_1^q$ and thus this main effect does not have a direct interpretation. However, the pre-post intervention main effect in the last column of the matrix does not get altered as within each unit the covariates are the same pre- and post-intervention and the point estimate of the pre-post intervention main effect variance of the estimates (that can be easily obtained from this centered design) is the same as the variance of the estimate had the non-centered $w_{h,i}^q$ been used.

We will not go into incorporation of time variant variables as this may happen causally from the intervention and either be in the casual pathway or in some settings create endogenicity problems. However, we suspect that if one is certain there will only small changes over time in covariates that are independent of the intervention that the above formulas are roughly applicable as well.
